# A System of Clinical Medicine

**Published:** 1843-07

**Authors:** 


### Art. XVI.

*A System of Clinical Medicine.* By R. J. Graves, M.D. M.R.I.A., Physician to the Meath Hospital, Dublin, &c. &c.—*Dublin*, 1843. 8vo, pp. 937.

In giving an account of the contents of this bulky volume it is unnecessary that we should enter into any lengthened introduction. The name and high reputation of its distinguished author are known to all our readers, and much of the materials of which it is composed has been already for some time before the public. The form in which they now appear will be found to differ chiefly in the bringing together and comparing of what had been before delivered at different times, in the suppression of some redundancies, and in the correction or illustration of the remarks from subsequent experience and accumulated facts. It is well known that from time to time several admirable clinical lectures by Dr. Graves, printed, as we are told, from notes taken by a shorthand writer, have been published in the London Medical Gazette and other periodicals. The first part of the work before us consists of these lectures or a selection from them arranged and corrected as we have stated above; the second part consists of detached essays upon various subjects here collected together from the Dublin Hospital Reports and the Dublin Medical Journal. It may admit of some question whether the author has done wisely in adhering so closely to the form in which his observations were originally given to the public, and it must be admitted that the work would have taken a more philosophical and systematic cast had a different plan been adopted. Still there is something in the, we had almost said desultory, nature of the clinical mode of conveying instruction which tends to bring the subjects of observation strongly before the mind and to impress them on the memory; and we must allow that Dr. Graves has in this manner succeeded in giving a graphic force to the views which he is desirous of illustrating, investing them with an air of reality, and adding much to the conviction of their truth and fidelity.

The first three lectures are introductory, and embrace the consideration of subjects which, however interesting in themselves and profitable perhaps to the student when delivered, might, we think, have been advantageously dispensed with in the circumstances under which the volume before us appears. The main value of the work is derived from its practical tendency, and we feel assured that few of those who consult its pages will be disposed to enter upon the theoretical questions propounded in the lectures referred to. The doctrines laid down in these introductory lectures are moreover such as many will be found to differ from, and although on the present occasion we intend to confine our remarks mainly to the far more important practical questions with which the succeeding portions are occupied, we are compelled in the outset to indicate our dissent from some of the opinions advanced by Dr. Graves, lest by our silence we should be thought to give them an approval which we cannot altogether bestow.

Briefly to allude to one point alone—we are by no means advocates for requiring from the candidate for medical diplomas and degrees a knowledge of the minutiæ of every science which bears in the remotest degree upon the theory and practice of medicine. We are quite aware that for the practical purposes of attendance upon and relief of the sick, it is especially the accomplished physician, taking the word in its most extended sense, who will be the real agent of good, rather than the chemist, the botanist, the natural philosopher, or even than the anatomist or physiologist; but we are not on that account to undervalue or set aside chemistry, natural history and physics, anatomy and physiology. To enter into this question would carry us far beyond the space which we can here devote to it, and we must leave this contest about the outworks, satisfied with guarding our opinions from misconception, and our silence from being construed into assent.

Fever. Lectures four to nineteen inclusive are devoted to this subject, and contain an exposition of the author's views upon several important points connected with the practical management of patients suffering under different forms of idiopathic fever. We must, however, in the outset guard the younger readers of Dr. Graves's work against too exclusive an adoption of certain of the special points of practice therein advocated. Fever is a very indefinite term, and there is perhaps no disease, assuming its various types to constitute varieties of one and the same general affection, which presents greater variety of aspect or requires a greater and corresponding variety of treatment. In giving this caution we are merely expressing what Dr. Graves himself would have urged had his lectures been addressed to those circumstanced as are the general community of medical practitioners. The typhoid fever of Dublin and the adjacent neighbourhood seems to be what is now known and described under the term maculated or spotted fever; in which, whatever other organs may be affected, the brain and cerebral system are often severely implicated. To this form of fever the observations of Dr. Graves more particularly apply, and in this form his opinions, we believe, will be found a valuable guide to practice. But we need do no more than appeal to the experience of the numerous intelligent practitioners allocated in the metropolis and other large towns, or scattered throughout the provinces, on this side of the channel, to show that the varieties of fever are as numerous perhaps as the localities in which it prevails. In many extensive districts of this country maculated typhus is unknown; in others it is of comparatively infrequent occurrence. In Liverpool and Glasgow and other large towns of the western side of the island the maculated form will probably be found prevailing, but we much question whether the central, eastern, and southern portions of the kingdom would not show other types of fever, in which the abdominal and pulmonary organs are much more affected than the brain, and the disease for the most part presents neither maculæ on the skin nor follicular disease in the intestinal mucous membrane. With these preliminary remarks, we proceed to draw attention to the treatment proposed and adopted by Dr. Graves in the severer forms of maculated fever.

The main features of his practice seem to be close and careful watching of symptoms, with the view not only of obviating the tendency to fatal or otherwise injurious effects, but also of anticipating and suppressing the outbreak of those local actions which are commonly observed to be the precursors and often also the immediate causes of mischief. Hence we have impressed upon us at the outset the advantage and indeed necessity of frequent visiting of fever patients by the physician, and of careful watching and nursing by competent assistants and experienced nurses. Directions are given to be on the watch for the slightest irregularity or discrepancy in the mode in which the different functions are performed, and especially those more immediately connected with the brain and nervous system.

It is sufficiently evident from the general tenor of the observations made by Dr. Graves that the chief danger in the fever which he has been accustomed to treat in the Meath hospital and in the city and neighbourhood of Dublin, arises from the affection of the brain. Yet we find him coming to very opposite conclusions respecting the nature of this state to those drawn by Dr. Clutterbuck and his followers :

"I spoke at my last lecture," he says, "of a man named Cassels, who died in the fever-ward with symptoms of cerebral excitement, and stated that I regretted having omitted to leech his head, and prescribe tartar emetic in the form of enema. Since that time we have had an opportunity of examining his body, and the results of the dissection are well worthy your attentive consideration. He was a young man of robust habit and apparently good constitution, and laboured under the ordinary form of maculated typhus. Shortly after his admission he was attacked with delirium, which was soon afterwards followed by coma and death. Now, suppose you were called to see a patient, not labouring under typhus, but exhibiting a similar train of symptoms, that is to say, violent delirium, accompanied by flushing of the face, suffusion of the eyes, headach, and a tendency to get out of bed, in fact, a state of furious excitement, requiring the restraint of a strait-waistcoat,—what idea would you be likely to form of the condition of the brain? If a patient of this kind had no typhoid symptoms, you would certainly say that he was labouring under meningitis or cerebritis; and if the case proved fatal, you would naturally expect to find lesions of the brain fully sufficient to account for all his symptoms. And you would in all probability find extensive thickening of the membranes of the brain, with subarachnoid effusion, or you would discover softening, increased vascularity, and suppuration of the encephalic mass. But here, a man in fever exhibits all the symptoms of cerebral inflammation; the cerebral affection runs on to a fatal termination with great rapidity; he dies comatose. And what do we find on dissection? Doubtful signs of congestion, and no distinct evidence of inflammation; a slight opacity of the arachnoid at the base of the brain, and about a teaspoonful of clear subarachnoid effusion. Now this is a point to which I would earnestly call the attention of every inquiring student. A patient, during the course of typhus, is seized with symptoms which are generally regarded as characteristic of congestion and inflammation of the brain; he dies, to all appearance in consequence of the intensity and violence of these symptoms, and on dissection little or no trace of cerebral disease is found. In the case under consideration the symptoms present were strongly indicative of congestion, if not of inflammation; and had the man been free from typhoid symptoms, you would expect to find decided traces of inflammatory mischief. This seems to prove that in the production of cerebral symptoms in typhus, some cause not to be recognized by the production of cerebral lesions, or in other words something beside mere congestion or inflammation, exists. I have now examined a great number of cases of this description, and the examination has brought home to me a strong conviction, that the delirium of fever depends upon something more than mere inflammation or congestion." (pp. 97-8.)

Now in these views of Dr. Graves we do not hesitate to express our decided concurrence, and we might appeal to the results of the practice founded upon either supposition as well as to the appearances on dissection to establish their correctness. Still in those cases, where the cerebral system is deeply implicated, the most frequent form of fever, as it seems, in Dublin, though of comparatively rare occurrence in some districts of this country, the danger to be apprehended is mainly from this source, and every precaution must be employed to guard against its occurrence, and every curative measure adopted to obviate its effects.

"What I wish to impress upon you is, that you should always anticipate the cerebral symptoms in fever. Never allow the cerebral symptoms to explode—watch the first scintillæ of cerebral excitement—repress the commencing mischief, and do not permit your patient to be overtaken by formidable inflammation of the brain. Every writer will tell you that when the patient's face is flushed, his eyes suffused, and when he complains of headach and intolerance of light, you should leech and blister his head, give him purgatives, tartar emetic, James's powder, and the medicines calculated to bring down cerebral excitement; but a careful and observant practitioner will anticipate all these symptoms, although there is as yet no particular flushing of the face, headach, or suffusion of the eyes; and though the patient is still quite rational, he will recognize threatening disease of the brain, and take proper steps to prevent its increase. Watch the functions of the brain attentively, and they will inform you in almost every case, of the approach of cerebral symptoms. You will find in patients who are about to have cerebral symptoms, a degree of restless anxiety, and a higher degree of energy than accords with their condition; and they either do not sleep at all, or their sleep is broken by startings and incoherent expressions. When you speak to a person in this state, he answers in a perfectly rational manner; he will tell that he has little or no headach; and were you to be led away by a hasty review of his symptoms, you would be very likely to overlook the state of his brain. If you inquire closely, you will find that he scarcely ever sleeps or even dozes, that he is irritable, excitable, frequently incoherent, and muttering to himself. Under such circumstances, although there is no remarkable heat of scalp, suffusion of the eye, or headach, I am frequently led to suspect the supervention of cerebral symptoms, particularly about the ninth or tenth day of the fever, (for it is generally about this period that cerebral symptoms begin to manifest themselves;) and whenever I observe these premonitory indications, I never hesitate in taking proper measures to anticipate the evil." (p. 86.)

"There is, on the other hand, an opposite state of the patient, which in like manner informs me that danger to the brain is at hand. In this case, the patient is almost continually sleeping. When you enter his chamber in the morning, and ask how he does, his attendant generally tells you that he has passed the night most favorably, and that he has slept without almost ever waking since your visit on the preceding afternoon. If he awakens to take drink, he quickly drops asleep again, and when you arouse him he looks rather heavy; there is some slight suffusion of the tunica adnata, and some appreciable congestion about the external parts of the face and head. Persons in this state, though apparently doing well, and even where they have been properly treated in the beginning, about the ninth or tenth day begin to rave, and exhibit undoubted proofs of congestion and excitement of the brain." (p. 87.)

In either of these states, Dr. Graves advises the immediate application of a blister to the shaven scalp, and considers that by this means he has the whole external surface of the head pouring out serum, or even suppurating, at the period when formidable cerebral affection would otherwise have set in. We believe that in the fever commonly observed in this country a less severe form of the same remedy, or an application which is less repugnant to the prejudices of the friends may suffice, but in this the practitioner must be guided by the character of the prevailing fever, as modified by local or epidemic influences.

A valuable symptom in indicating the existence of insidious disease of the brain in fever is the occurrence of vomiting. Nausea, vomiting, and diarrhœa are, however, equally accompaniments of those forms of fever in which the gastric mucous membrane is implicated. It becomes of importance therefore to be able early to detect the difference between the vomiting which arises from either cause. Dr. Graves expresses the opinion that vomiting and purging setting in at the commencement of fever usually indicate cerebral affection. This view is in accordance with what is observed in injuries of the brain from other causes, and there are few who have been called upon to witness a severe epidemic of scarlet fever, who can have failed to remark that those cases in which vomiting came on early, proved to be of the most serious description, in consequence of the head affection. In the epidemic which has recently been so fatal in some parts of England, there has been frequent occasion to verify the correctness of this observation.

Cerebral vomiting, as it has been already stated, commonly sets in

"At a very early period of the disease, perhaps on the first or second day, and is seldom accompanied by the red and furred tongue, the bitter taste of the mouth, the burning thirst, and the epigastric tenderness, which belong to gastro-enteric inflammation. There is also another source of diagnosis, but of a less valuable kind; and this is founded on the results of treatment. Gastro-enteric vomiting and diarrhœa are relieved by leeching the belly; but I need not tell you that this mode of treatment can have no effect on the vomiting and purging produced by cerebral disease. There is also another means of distinguishing; the vomiting and diarrhœa which results from gastro-enteric inflammation is never accompanied by such copious discharges of bile as that which depends on disease of the brain. In diarrhœa from derangement of the brain, the quantity of bile passed is very remarkable; and it is equally curious, that when vomiting follows derangement of the cerebral circulation in ordinary cases, and without fever, bile is thrown up in very large quantities. This is frequently observed in persons who become sick from swinging or sailing. In such instances, a larger quantity of bile is vomited than could occur from mere gastric irritation. Now in the commencement of cerebral disease, where congestion or inflammation is present, one of the first symptoms is copious vomiting, and purging of a bilious character." (pp. 94-5.)

There is room, however, for caution against coming to too hasty a decision where the discharges are of a bilious character, since many cases of what are termed bilious fever and bilious indigestion are characterized by early vomiting, and copious flow of bile; and where the vomiting in fever is of a bilious character, there will be a bitter taste in the mouth, and the tongue will be found to be furred pretty much the same as when the symptoms in question are dependent upon gastro-enteric disease. In short, cerebral irritation of whatever description, very generally gives rise to vomiting and purging, which may or may not implicate the secretions of the liver. A similar effect also occurs from gastric irritation, and the chief points of distinction between them are the presence of pain at the epigastrium, and the peculiar red furred tongue which attend the latter.

Dr. Graves points out a peculiar state of the respiration connected with cerebral excitement in fever which when present affords perhaps a symptom of less ambiguous character.

"Now there is one symptom connected with cerebral excitement in fever, which is well worthy of your notice, as its existence is often sufficient of itself to give timely intimation of the approach of irritation or inflammation of the brain. This is the state of the respiratory function. In fever, the breathing will often announce the approach of cerebral symptoms for days before their actual occurrence. When, in cases of typhus, you find the patient's breathing permanently irregular, and interrupted by frequent sighing, when it goes on for one or two minutes at one rate, and then for a quarter or half a minute at another rate, you may rely upon it that sooner or later, an affection of the brain will make its appearance. You will frequently observe the same kind of breathing preceding attacks of apoplexy and paralysis, and indeed it was the occurrence of this symptom, in these and other cases in which the functions of the brain were deranged, that first drew my attention to this kind of breathing. The first time it engaged my attention was in a remarkable case of an apoplectic nature, which I sat up a whole night to watch. On recollection, I found that I had frequently observed an analogous state of the respiratory function in fever, on several occasions, although its connexion with excitement of the brain had not struck me before. I speak here of irregularity of breathing, independent of any pectoral affection. But when the patient breathes in a permanently irregular manner, at one time at a certain rate, and at another at a different rate, when his respiration is suspicious [suspirious?] and heaving without any disease of the chest or great debility, you will have some grounds to suspect the existence of cerebral derangement. I am in the habit of calling this kind of breathing *cerebral respiration*, because my experience has told me that it is almost invariably connected with oppression and congestion of the brain. To recapitulate. When you find a patient in fever lying constantly awake, or when, on the contrary, you find him continually slumbering, when there is a certain quickness of manner and irritability, and when the cerebral respiration has been noticed for some time, without any concurrent debility or pulmonary disease, under such circumstances, you may, in cases of maculated typhus, predict the approach of cerebral symptoms; and the period about which they generally manifest themselves, is the eighth, ninth, or tenth day." (pp. 87-8.)

We have before urged the importance of anticipating the outbreak of the cerebral affection, and given at some length the directions which Dr. Graves lays down for effecting this purpose. It now remains to notice the remedial measures which he recommends when cerebral excitement is actually developed and threatens the safety of the patient. Tartarized antimony has been frequently employed in fever and in some epidemics with excellent effect. In others, it has on the contrary seemed to prove not only inefficacious but decidedly prejudicial. In these cases however, the remedy has been usually administered in a different stage of the fever and with different views. It is Dr. Graves's peculiar merit that he has exhibited the tartar emetic with success under circumstances of almost hopeless nature, and that he has been led to the practice by what we believe to be a correct view of the pathological state under which patients so circumstanced suffer. It appears sufficiently evident that the same or similar symptoms may arise in very opposite states, and that the resemblance is often so great, that careless, superficial, or biassed observation may readily lead into error. Who is not aware, that giddiness even to falling, is not unfrequently owing as much to a state of debility as to one of plethora, requiring also a diametrically opposite mode of treatment for its relief? The writings of the late Dr. Gooch afford evidence of a similar character in regard to other affections. Dr. Graves extends this principle to fever, and shows that delirium, sleeplessness, and other symptoms usually considered to be indicative of vascular disturbance in the brain, are not necessarily dependent upon inflammation, and require for their management a corresponding mode of treatment.

The author's claims to originality in proposing the peculiar treatment by which he endeavours to meet this condition have, it seems, been disputed. However otherwise he may feel himself aggrieved, Dr. Graves has some cause for satisfaction with the testimony thus afforded to the usefulness of the treatment proposed. Where a discovery of whatever nature is of no value, few are disposed to say much about it, and the reputation for that which is of doubtful or injurious character is generally left in the indisputed possession of those to whom it may belong. Our present object, however, is to endeavour to appreciate the merits of the practice followed by Dr. Graves, not to assign to him the precise degree of originality which he is entitled to claim, for proposing and carrying it into effect.—Now, when we meet with a fever patient who on the nineteenth day of the fever has had no sleep for many days and nights, and is found to be in a state of mental incoherence, raving, tremor, and subsultus, with a failing pulse, we must allow that there is cause for the most serious alarm. Let us see what Dr. Graves proposes under such circumstances:

"I remember well the time," he observes, "when a patient so situated would have been again purged, his head would have been shaved, a few leeches applied to the temples, and a blister to the nape of the neck, while perhaps wine and musk would have been exhibited internally. How many persons have I seen so treated by the most eminent physicians, and how unsuccessful was the practice! To have talked of giving opium under such circumstances, and when the marks of cerebral congestion were so evident, would have been regarded as absurd; my experience on former occasions, however, determined me to give opium, and, as the danger was imminent, I gave it boldly. To the eight-ounce mixture, with four grains of tartar emetic, we added one drachm and a half of laudanum; of this he took one ounce every second hour, from eight in the evening until he had taken five doses. This produced copious sweating; the skin became cooler, he raved less, but still no sleep; at four on the following morning, his pulse became 70, and respirations tranquil; he got twenty drops of Battley, and at half-past five in the morning, twenty-five drops more. He had now taken within a short time, about one drachm of laudanum, and forty-five drops of Battley, combined with nearly three grains of tartar emetic. He was tranquil, but did not close his eyes, and muttered occasionally; subsultus less. His pupils now became more and more contracted, his eyes less expressive and duller, and when I came at eight in the morning, he was evidently deeply narcotised, although not yet asleep. I thought that all was lost; but still, observing the respiration to be tranquil, and the pulse regular, I indulged a faint hope that sleep might still supervene. His eyes now became still more inexpressive, the lids gradually closed, his breathing became prolonged and deep, and at half-past eight he was buried in a profound and tranquil sleep, which continued for nine hours, when he awoke, spoke rationally, said he had no pain in the head, took some drink, and fell asleep again. Next morning not a single symptom of fever remained." (pp. 150-1.)

The condition to which several of the fever patients treated by Dr. Graves were reduced seems to be analogous in many respects to that which occurs in delirium tremens. It is here that the combination of tartar emetic and opium is so strenuously recommended, and seems to have proved in the cases related so beneficial in its effects. In the earlier stage of fever, when inflammatory or congestive symptoms make their appearance at the outset of the attack, Dr. Graves is disposed to meet them with the lancet, leeches, purgatives, cold applications, and tartar emetic.

"Here," he observes, "the lancet and tartar emetic are our best opiates, our best restoratives of tranquillity and sleep. As the fever progresses, and when we have arrived at a more advanced stage of the disease, when maculæ makes its [make their] appearance on the skin, and symptoms of general debility announcing the typhoid type begin to predominate, then we must proceed with more caution, even though our patient is totally deprived of sleep and is violently delerious. The lancet cannot now be resorted to; leeches, indeed, may be applied, but their effects must be carefully watched, as the patient will not bear copious depletion of any sort; tartar emetic may, nevertheless, still be given boldly, and will be found to answer our expectations. But if we have to contend with want of sleep and delirium at a still more advanced period of fever, we now often recognize that very combination of symptoms, the union of general debility, and cerebral congestion, which in certain varieties of delirium tremens we have seen so successfully treated with tartar emetic and opium; who will refuse to acknowledge the similarity between these cases of fever, delirium, and many varieties of delirium tremens? Are there not in both, the same tremor and subsultus of the extremities; the same trembling of the tongue when the patient endeavours to put it out; the same starting and sleeplessness; the same rambling, delirium, or incoherence, combined nevertheless with the power of answering rationally when spoken to; the same character of the mental wandering, for in both they are extremely apt to rave as if employed in their ordinary occupations, and as if surrounded with their usual associates; in short, can any greater resemblance exist between two diseases arising from the operation of remote causes so different? We need not, therefore, be surprised, at finding the same treatment applicable to both." (pp. 155-6.)

Tartarized antimony, as we have before observed, has been frequently employed in fever cases; the peculiarity of the treatment pursued by Dr. Graves, arises from the manner in which this remedy is given. In the earlier stages the doses are larger than those commonly employed by other practitioners, and are repeated at shorter intervals; in the later stages the antimonial is given in combination with opium, as we have just shown. We have one observation to make upon this method of treating fever cases. It may admit of question whether the later symptoms simulating those of a congestive or inflammatory condition of the brain are not occasionally owing to the early treatment of the attack being conducted too much on the antiphlogistic plan? May not the *nimia diligentia medici* in the use of antimonials in the early stage, sometimes bear its part in giving rise to the apparent cerebral excitement developed in the future progress of the disease? It is well known that the active employment of depletion and antimonials in cases of disease purely inflammatory or in inflammation as it occurs in certain conditions of the system, will often give rise to an irritative action of the vascular and nervous systems and determination to the diseased part, apparently calling for the continuance or repetition of the same measures, but to be met only by others of a very different character. There seems no sufficient reason why something of this kind may not also arise in severe fever; but however this may be, we are ourselves disposed to be sparing in the employment of antimonials wherever fever assumes or threatens to assume the typhoid type. We do not make these observations in disparagement of the treatment pursued by Dr. Graves. The fever which he has been accustomed to treat may, and probably does require a different method of management to that required in the fever we are accustomed to witness. There may possibly be more tendency to an inflammatory type at its commencement, and the local complications may at this period be of a more active and threatening character. To meet the almost desperate circumstances of the latter stage which have been above detailed, the use of tartar emetic and opium in the manner recommended should be had recourse to wherever they occur, as we know of no means more likely to prove of service in such cases, and certainly of none which come to us so strongly recommended.

These observations on fever have extended to a greater length than we had intended, but before concluding them we must briefly notice one or two other points of some practical importance.

No one can question the utility of cold lotions when the head is much affected; but, as Dr. Graves elsewhere observes, there is a vast difference between a thing being done and its being well done. In the very imperfect manner in which cold lotions are generally employed, the effect is, we firmly believe, worse than useless, the congestive state of the brain being often augmented instead of relieved by them. Strongly impressed with the mischiefs resulting from the partial employment of cold applications to the head, Dr. Graves recommends lotions of warm vinegar and water instead, and we can ourselves testify to the great relief and advantage which may be thus obtained. In cases where it is desired to obtain the counter-irritation of a blister without its debilitating effects, a circumstance which often arises in the latter stage of fever, and in cases where there is much debility, Dr. Graves recommends that the blister should be kept on from four to six hours only, neither removing the cuticle nor letting out the effused serum when the blister is removed, but dressing it at once with spermaceti ointment. This method of blistering is especially applicable when the chest is affected, either in the advanced stage of fever, or in some forms of pulmonary disease, and especially among children. The pain and local irritation is much less, there is little or no irritative fever, and less risk of affecting the urinary organs, while in children the danger of inducing troublesome sloughing ulceration is avoided. The practice is not new, but deserves more attention in general practice than it now receives.

The employment of wine in fever requires much consideration, both as to the time when, and the quantity in which it is advisable to give it. Dr. Stokes has recently attempted to derive indications for its use from the characters of the cardiac sounds and impulse. Without entering upon a criticism of the theoretical views advanced by Dr. Stokes, we may briefly state that Dr. Graves, admitting the fact that the heart in many cases of typhus becomes debilitated, and that this debility is manifested by a corresponding change in the nature of the sounds and impulse afforded by the organ, fully agrees with Dr. Stokes, "*that in the diminished impulse, and in the feebleness or extinction of the first sound, we have a new, direct, and important indication for the use of wine in typhus fever.*"

In one of the lectures devoted to the subject of fever, the eighteenth, we have an account of an epidemic occurring in Dublin in the year 1826, attended with jaundice, which Dr. Graves terms yellow fever. A comparison is instituted between this epidemic and the yellow fever of

Gibraltar and the south of Spain of the year 1828. There appears to have been some essential differences between the two, and the propriety of applying the term yellow fever to the Dublin epidemic or rather to those cases of it in which jaundice formed one of the complications is very questionable.

Scarlet fever. This is not the subject which follows next in the order of succession in the work before us, but as it may be more conveniently considered in connexion with the preceding observations on general fever, and as the method of arrangement followed by the author, if indeed any methodical arrangement at all have been adopted by him, is not easily made out, we shall not hesitate to transpose the subjects as may seem most fitting or most convenient for our purposes. The thirty-fourth lecture, in which the account of scarlet fever finds its location, was delivered during the session of 1834-5, and the observations chiefly refer, we presume, to the scarlet fever then or for some years before prevailing. To these however are appended letters from various practitioners, dated 1842; a wider range must therefore be allowed, embracing other epidemics of scarlet fever in addition to those to which they are more immediately applicable. We are at some loss to characterize the features peculiar, if any such there be, to each epidemic, and we do not always find a clue to assist us in so doing, a somewhat singular omission, after the remarks with which Dr. Graves has prefaced his account. The scarlet fever of the year 1834 assumed a much more serious and fatal character than what had been before observed during the present century, and Dr. Graves describes three forms under which the more severe cases presented themselves. In the first of these there was, with the usual fever and sore throat, violent congestion of the cerebral organs at the outset, with convulsions and apoplectic coma on the first or second day, attended in the worst cases with general and intense cutaneous efflorescence. In this form when the tendency to the head was early and strongly manifested, the patient was seldom saved, though sometimes very active depletive measures seemed to relieve the brain and the result was then favorable. Cases of this form we remember to have observed on this side the channel in the summer of 1832, during the prevalence of the epidemic cholera, and in more than one instance a fatal termination was produced by the first shock of the attack, within a few hours of the sinking under the disease. In one case, occurring in a fine young woman, the whole surface of the body after death was of an intense purple red; in another, that of a child, the attack was almost immediately fatal, some very imperfect traces only of eruption having transiently appeared.

In a second form of the disease noticed by Dr. Graves there were the usual symptoms of severe eruptive fever, attended from the onset with severe headach and spinal pains and great irritability of the stomach and bowels; nausea, vomiting, and bowel complaint being among the very first symptoms. Recently-secreted bile was thrown up in large quantities, and abundance of bilious and mucous discharges with some fecal matter passed from the bowels. These discharges from the stomach and intestinal canal afforded not the slightest relief either to the fever or the headach, nor did they seem to interfere with the formation and progress of the eruption; and during their continuance there was not the slightest tenderness of the epigastrium nor of any part of the abdomen, the belly, on the contrary, becoming fuller and soft. It is impossible not to recognize in this irritable state of the stomach and abdominal viscera the characteristics of cerebral irritation and congestion before pointed out under the head of Fever. This form was also attended with much disturbance of the circulatory system, the pulse being rapid and the temperature of the surface elevated. Depletion was ill borne in cases of this description, and Dr. Graves draws the inference that

"The derangement of the brain and nerves depended on something more than the violence of the circulation, and originated in something altogether different from mere cerebral inflammation or congestion. What that something was," he continues, "I cannot even conjecture; but it was probably the result of an *intense poisoning of the system by the animal miasma of the scarlet fever.* Every tissue of the body seemed, if I may use the expression, equally sick, equally overwhelmed, and it is probable that the capillary circulation in every organ was simultaneously deranged. It was not gangrene of the throat which proved fatal, *for in this form it never occurred;* it was not inflammation of any internal viscus, for such was not found on post-mortem examination of the fatal cases; but it was a general disease of every part." (p. 506.)

Cases very similar to the above we have had occasion to witness during the past year, and in one of these the symptoms were almost such as might, under other circumstances, have led to the suspicion of poisoning.

The remarks of Dr. Graves on another state of things occurring in many patients, which he says required to be carefully distinguished from that just described, and where the disease was evidently attended with an inflammatory state of the constitution demanding energetic measures, but ill agree with the views respecting the epidemic constitution with which he commences the subject under consideration. In such cases the early symptoms were severe and the throat very sore, but the eruption was not quite so perfect in its appearance nor was the pulse so rapid. Bleeding and leeching were in such instances borne well and followed by almost immediate relief.

The third form described by Dr. Graves was ushered in by the usual fever together with sore throat and slight headach, and followed by a regular and moderate amount of eruption; but when all danger seemed to be over and convalescence apparently commencing, about the eighth or ninth day, slight restlessness and morning fever were observed, which were followed by an irritable appearance of the nostrils, return of sore throat, heat of skin, sudden and great prostration of strength, and painful swellings in the region of the parotid and submaxillary glands. These swellings rapidly increased and were attended with ill-conditioned viscid fetid secretions from the nostrils, mouth, and throat, the mucous lining of these parts being inflamed and tumefied. Under such circumstances the fever rapidly increased and assumed the typhoid character, the fatal termination following after much suffering. It is remarkable that in not any one of the three forms of the disease here noticed, formidable as they were, does the sore throat appear to have been of primary or essential importance.

A good illustration is afforded by scarlet fever of a general proposition or law laid down by Dr. Graves, "that in both acute and chronic diseases *a constitutional affection may display its existence by only one or*

*two of the numerous symptoms which usually accompany it.*" "This occurrence," he observes, "seems more frequent in the case of diseases produced by contagion and morbid animal or vegetable poisons, than in the case of maladies generated by causes developed in the system itself." (p. 538.) The "scarlatina simplex, nulla comitante cynanche" of Cullen, described also by Sydenham, and the "rubeola sine catarrho" of Bateman, are examples familiar to all, while the sore throat so frequently observed to prevail during epidemics of scarlet fever is probably another illustration of the same principle. It will be recollected that the desquamation of the cuticle occurring at the close of scarlet fever has been attributed to the effects of the inflammatory process going on or supposed to be going on in the skin during the appearance of the efflorescence, but Dr. Graves shows that the desquamation occasionally takes place where there has been no eruption, and he also mentions a case in which a young lady, who after a constant attendance on her sisters during their illness from scarlet fever, but who had herself apparently resisted the infection, was subsequently attacked with the peculiar anasarca so well known as one of the sequelæ of scarlatina.

Some remarks are made on the occurrence of albuminous urine in the dropsy connected with scarlatina, which is considered by Dr. Graves, in accordance with the opinion expressed by Dr. Blackall, as indicating merely a peculiar inflammatory condition of the whole system unconnected with degeneration of the kidneys. In support of this view he gives the particulars of a case in which, after death, the kidneys were found to be perfectly healthy, although the urine had been highly albuminous throughout and the dropsical swelling extreme. Dr. Graves therefore considers this state of urine and the morbid condition of the kidney with which it is so commonly connected to arise from different causes often co-existing in chronic dropsy but not necessarily dependent the one upon the other. He consequently regards albuminous urine as a sign of Bright's kidney, but not as its result. In the anasarca following scarlatina Dr. Graves highly recommends the employment of the hydriodate of potash in addition to the usual means, a remedy the good effects of which in dropsical swellings, we may add, are not confined to the forms here spoken of.

INFLUENZA. We do not perceive that Dr. Graves has added much to our knowledge of the causes of those epidemic visitations of catarrhal fever, so many of which have occurred within the last twelve or fourteen years. The exclusion of vicissitudes of temperature, peculiarities of situation, supposed moist or dry states of the atmosphere, the prevalence of certain winds, with many more presumed sources of epidemic disease, form the chief agency in the generation of influenza, is no more than what has before been recognized by others, and after all affords but a negative characteristic of its etiology. To say that "it is probable that influenza may depend chiefly on telluric influence, upon some agency connected with variations in the physical conditions which operate on the external surface of our planet," is only another mode of expressing our entire ignorance, which it would be better at once to acknowledge in so many words and without attempting to involve it in the language of useless and groundless conjecture. But whatever may be the cause to which influenza owes its origin, there can be no doubt that from the severity and fatality of some of its recent visitations as well as from their wide spread and almost universal prevalence, the best mode of management of those suffering under this affection becomes a subject for serious consideration.

Notwithstanding the expectations formed by some practitioners from depletive measures, which expectations, by the way, seem to have been more sanguine amongst our countrymen on the opposite side of the channel than they were on this, there is little doubt that the general impression now is that bleeding, leeching, &c. in the greater number of cases, and unfortunately also in the more severe ones, were too often worse than useless. Dr. Graves, although he thinks that in some instances venesection was of great service even when the pulse was natural, expressly states that in others it could not be borne to the smallest amount when the pulse was hard and wiry, and remarks that he never saw any disease in which the pulse formed so bad a guide as to the propriety of venesection as the present epidemic, (that of 1837.) Upon the whole he seems to consider the disturbance of the circulation in influenza to be connected with irritation of the nervous system rather than with any inflammatory state of the constitution in general, an opinion which agrees we suspect with that of most well-informed and reflecting physicians. He subsequently observes that bleeding, unless employed within the first twelve or twenty-four hours, is likely to do as much or more harm than good; and that on the second or third day, except to relieve congestion of the lungs, it seems inadmissible. Leeching over the hollow of the neck just above the sternum, aperients, confinement to bed, and sudorifics, are the remedies which he chiefly recommends when the first twenty-four hours have passed over, adding opiates in combination with nitre and tartarized antimony after the antiphlogistic remedies have been persevered in for a day or two. Blisters, according to the experience of Dr. Graves, afforded no relief either to the dyspnœa or to the pulmonary symptoms in general. Fomenting the trachea and chest with hot water seemed to be more serviceable, a practice which is also applicable to many other diseases where the air-passages are affected.

There is no novelty, it will be observed, in this mode of treatment, and little difference from that generally followed in this country, and we have been induced to give the foregoing brief summary of the practice of Dr. Graves in this affection, only because in diseases of such extensive prevalence as influenza it is desirable to be acquainted with the methods of treatment adopted by those practitioners whose means of observation and sound practical judgment render their opinions upon almost any subject worthy of notice.

Bronchitis. The miscellaneous nature of the contents of the work under notice must necessarily give the same character to the remarks which we feel called upon to make. Having therefore brought the subjects of scarlet-fever and influenza into juxta-position with general fever to which they are nearly allied, we now retrace our steps and take up the consideration of bronchial inflammation, which in the "system" follows immediately after fever. Dr. Graves is of opinion that catarrhal disease, taking its rise from exposure to cold, may be equally occasioned by direct impression of the inspired air on the bronchial membrane as by the secondary effects of a chill on the surface, and though there are evident provisions calculated to act as a check to the mischiefs which might result from the respiration of air greatly reduced in temperature, we cannot doubt that bronchitis does occasionally arise in this way. The stethoscopic signs of bronchial inflammation are dismissed in few words, Dr. Graves contenting himself with pointing out the difference between those of the cases in which the larger bronchi alone are concerned, and those where the minute tubes are implicated. In the former the sounds under the stethoscope are few in number, of a deeper tone when dry, or when moist the bubbles large, unequal, and scattered; in the latter many sounds may be perceived together in a limited space, which are small, wheezing, dry and sharp, or very minute and evidently moist. We agree with Dr. Graves and Dr. Latham that the burdening of the memory of the young auscultator with names for so many different varieties of râle tends only to produce confusion in his mind, and it may be added that if every modification of sound appreciable to the practised ear is to receive its appropriate appellation and definition, the terms hitherto recognized are altogether inadequate, and might be multiplied almost indefinitely.

To confine our remarks to bronchial inflammation, the main object is to ascertain its locality and extent, whether the larger or smaller tubes are affected, and whether the secretion is greatly or at all increased. Among the cases which Dr. Graves selects to illustrate by his observations, is an instance of chronic bronchitis with an exacerbation from a fresh attack of cold. This is summed up as follows: "Extensive bronchial inflammation with copious expectoration unaccompained by fever, and occurring in a debilitated constitution." The only stethoscope phenomena were extensive minute and moist bronchial râles. In such a case depletion, either by lancet or leeches, is out of the question, for the temporary relief afforded by its employment is rapidly followed by an increase of the already abundant secretion and consequent severity of the symptoms. Dr. Graves especially recommends a combination of nitre, tartar emetic, and camphorated tincture of opium in some mucilaginous or demulcent fluid, with an active rubefacient to the chest. Where bleeding and leeches are contra-indicated, Dr. Graves gives the preference to the combination of nitrate of potash and tartarized antimony over all other remedial agents. In the subsequent steps of the treatment, though occasion arises for many judicious remarks adapted to a class of intelligent pupils, there is nothing which is not already well known to the profession. After relief has been obtained, pectoral mixtures of more stimulant character are recommended. Alkalies are praised as productive of good effects in cases of pulmonary irritation generally, an observation which we have often had occasion to verify; and when the cough becomes entirely chronic, the *mistura ferri comp.*, in smaller doses however than is usually given, combined with tincture of henbane, is much recommended. Dr. Graves remarks that excessive secretion from the lungs may often be diminished by a powerful hydragogue cathartic, and when the patient is sufficiently strong to bear it, such practice may occasionally be adopted with striking benefit. "This, however," he adds, "will require both judgment and discretion, and it should be borne in mind, that, in the majority of cases, there are many circumstances which contraindicate their employment." We will add the further caution that although a cathartic will often relieve chronic cough attended with copious secretion from the bronchial membranes, considerable discretion should always be used in the employment of purgative medicines after inflammatory affections of the lungs, as we have had occasions to witness more than once a relapse induced, unless we are greatly mistaken, by the operation of the cathartic.

In chronic bronchitis unaccompanied by fever, remarkable dyspnœa, or acceleration of the pulse, and when the secretion is copious, Dr. Graves has often experienced good effects from emetics; in other cases he recommends the use of sulphur, which he was led to employ from observing the benefit derived in cases of chronic cough and congested bronchial membrane from sulphuretted mineral waters. We have witnessed the good effect of sulphur in chronic catarrh in a case in which the remedy was used for a different purpose; and it seems to deserve a more extensive trial of its power than it has yet met with. Dr. Graves combines cream of tartar with the sulphur, partly with the view of tempering its stimulant properties, and partly to assist in determining to the kidneys in accordance with the axiom of Baglivi: "In morbis pectoris ad vias urinæ ducendum est."

Among the effects of bronchial irritation alluded to by Dr. Graves, there is related a case of asthma which gives rise to some excellent remarks on the use of counter-irritant remedies in that disease. These are recommended to be applied not merely over the chest, but to the nape and along the sides of the neck over the epigastrium, and in the course of the cervico-spinal and pneumo-gastric nerves generally. Various stimulating liniments and like applications have been at different times proposed and lauded for their effects in bronchial and other pulmonary affections, such as the celebrated liniment of St. John Long, Dr. Granville's ammoniated liniment, and others. The first of these has been analysed by Dr. Apjohn, and found to consist of acetic acid, spirits of turpentine, and two animal matters, the one containing azote, the other not. Dr. Graves is inclined to think that the spirit of turpentine exercises something more than a mere counter-irritant action, and proposes the following formula as an imitation:

Strong acetic acid, ʒss.
Spirit of turpentine, ʒiij.
Rose water, ℥iss.
Essential oil of lemon a few drops.
Yelk of egg, sufficient to suspend the turpentine.

There are some observations on cough and the various causes giving rise to it which are well worthy of attention, and may find their place here. Pulmonary irritation from diseased states of the lungs is of course the most obvious of these causes, but the specific source of the irritation is often involved in obscurity. Dr. Graves gives a remarkable instance in which the cough depended upon tape-worms, the patient having been cured by the prescription of an old woman (castor oil and turpentine given for an attack of colic,) after he himself and another physician, treating the case upon erroneous views of its nature, had exhausted their ingenuity in vain. Hysteria, a syphilitic taint, gout and rheumatism, scorbutus and scrofula may thus all give rise to irritation of the pulmonary organs and excite cough, which in each instance, will require a plan of treatment varied according to the peculiar source from which it derives its origin. We remember some years ago to have observed that very many of the cases of rheumatism occurring during a particular season were closely connected with bronchial disease, the usual disposition of the heart to suffer having been apparently transferred to the pulmonary mucous membrane. The ascertaining of these constitutional complications, or the specific character of the local disorder derived from them, is a point of the highest practical importance, since the very same remedial measures may prove curative, inert, or even fatally injurious, according to the source from which the symptoms take their rise. Thus in the case referred to in which the cough was dependent upon a sympathetic pulmonary irritation, excited by the presence of tape-worm, bleeding, leeches, blisters, and tartar emetic were employed without other effect than the injurious one of debilitating the patient. Antispasmodics and narcotics proved, to say the least, inert, while the symptom was at once removed together with its cause by the empirical dose administered for an attack of colic. Again, depletion would prove injurious in pulmonary irritation connected with hysteria, mercury in that arising from scrofula, while where there is a syphilitic taint this latter remedy has been observed to produce the most beneficial effects under circumstances of very threatening character.

LARYNGITIS. The next lecture is devoted to a case of pleuro-pneumonia proving fatal with an acute laryngeal attack and gangrene of the lungs, and to some remarks on pleuritis and pneumonia, and especially with reference to the occasional absence of expectoration in the latter disease from its commencement to the period of complete resolution. Dr. Graves makes the following observation on laryngitis, which, as assisting in the formation of a correct prognosis, is perhaps worthy of being recorded: "*In the aged it is accompanied by considerable fever, and, what you would suppose likely to give relief, copious expectoration, evidently derived from the larynx itself; and yet I do not recollect that I have seen an attack of this kind that did not terminate fatally.*" (p. 252.) Another form of laryngitis which pretty uniformly proved fatal, that which occurs in scrofulous constitutions, is alluded to in a subsequent lecture. This form, though commonly more or less of a chronic character, when it is termed laryngeal phthisis, is occasionally observed to run its course with great rapidity. Laryngitis, therefore, arising in a scrofulous constitution, or in a constitution debilitated by any cause, whether it assumes the acute or chronic form, should always be most carefully watched. In the chronic form, and especially when there is no evidence of scrofulous deposition in the lungs, Dr. Graves recommends the application of leeches in small numbers every second or third night, if there is much tenderness on pressure or motion. If there is no tenderness of consequence he advises nitrate of silver or sulphate of copper in solution in the proportion of ten grains to the ounce, to be applied by means of a sponge fastened to the end of a quill to the excoriated and inflamed part of the fauces. The object here is to change the action of the mucous membrane of the pharynx, fauces, and entrance of the larynx, and subsequently, by extension to the neighbouring parts, of the whole inner surface of the larynx itself. With these measures, counter-irritation and the other remedial agents usually employed may be had recourse to according to the varying circumstances of the case.

Hemoptysis, pulmonary apoplexy. As there are two sets of vessels from which the blood sent to the lungs is derived, the bronchial and the pulmonary, so also are there two sources from whence hemoptysis may derive its origin. Now, as is observed by Dr. Graves, the bronchial artery destined to supply that blood which is necessary for the nutrition of the pulmonary texture is small, and its blood arterial or red, while the pulmonary artery on the contrary is large and transmits the whole of the dark venous blood of the system from the right cavities of the heart through the lungs. It is sufficiently obvious therefore that the danger to the patient, and consequently the importance of the symptom, will be materially modified by the source from which the blood expectorated or poured out into the pulmonary structure is derived. Dr. Graves, from considerations arising out of the minute anatomy of the lungs, which it would be impossible to enter upon here, endeavours to show, " that when hemoptysis, from the engorgement of the system of the pulmonary artery, takes place, it is in consequence of the direct effusion of blood from the branches of the pulmonary artery, which ramify on the air-cells, and that the blood expectorated on such occasions has nothing to do with the bronchial mucous membrane, or bronchial arteries," (p. 261 ;) and further that the blood may escape also into the inter-vesicular pulmonary tissue, where, having no exit like the portion which is thrown out into the air-cells, it must remain. The union of these two forms of effusion from the minute branches of the pulmonary artery constitutes the disease termed pulmonary apoplexy; but it is important to be aware that the affection characterized by all its essential symptoms may occur without hemoptysis, an instance of which is quoted by Dr. Graves from the Dublin Medical Transactions. Are there any sufficient characteristics by which it can be ascertained whether the blood in expectoration is derived from this formidable source, or whether it comes from the bronchial artery ? Let us see what Dr. Graves himself says upon this point :

"In the first place the blood (from the pulmonary artery) is black, as you can perceive when it is spit up. It is also clear, that if this blood be detained for some time in the air-cells and bronchial tubes, it will become coagulated, and be spit up in clots. Many of the worst cases of spitting of blood are attended with this symptom ; and it is not [?] a mistake to suppose, as you see it mentioned in books, that blood expectorated from the lungs should be florid and frothy. You are told, gravely, that you can distinguish blood discharged from the stomach from that which is discharged from the lungs, by the difference of its colour, consistence, and the presence or absence of air-bubbles. No, gentlemen, you cannot. If you see blood spit up which is dark and coagulated, and, from stethoscopic examination, have reason to think that it comes from the lungs, you will be convinced that the effusion is from the pulmonary artery. I do not mean to say, that when blood comes from the pulmonary artery it is always black and clotted ; but I assert that it is so in a great majority of cases; and in many cases of pneumonia we find the sputa partake more of the venous than the arterial character, a circumstance which indicates its formidable source. It is obvious that the blood spit up in those cases may also have a florid tinge, where it has been imperfectly aerated by the imperfect action of air bubbling through it before it is expectorated. There are some hemorrhages, also, from the bronchial artery, which are very copious; but, generally speaking, where there is much cough, constriction of the chest and fever, it is the bronchial mucous surface which is affected ; and the spitting of blood which, in such cases, comes from the bronchial arteries, is but scanty, and is seldom dangerous. The blood will be found to be effused from small spots, as in epistaxis, and the quantity is generally small. You will, however, sometimes find an instance of a person spitting up, very copiously, blood of an arterial colour; for it must be borne in mind, that a very small surface of mucous membrane may often bleed most copiously, as is seen in some cases of epistaxis, when the blood issues from an insulated and small spot. Such cases of copious bronchial hemorrhage occur occasionally, are unconnected with bronchitis, and depend on some peculiar hemorrhagic tendency." (p. 263.)

The chief source of the immediate danger in cases of congestion of the pulmonary artery leading to hemoptysis, is not then from the amount of blood lost to the system, but from the plugging up and obliterating of the ultimate divisions of the air-tubes by the blood effused into and around them. This is important to be borne in mind as a guide to practice, since no hesitation need be experienced, at least in the outset of the attack, in attempting the relief of the congested state and the consequent arrest of the further progress of effusion by copious depletion. The effusion from the branches of the bronchial artery, on the other hand, is seldom extensive, and is derived, Dr. Graves thinks, in phthisical cases in which it is of such frequent occurrence, rather from the congested state of the bronchial membrane so commonly connected with that affection, than from ulcerative action implicating the vascular tissue. We regret that we cannot follow the author throughout the many important questions which are here brought before us, but we must rest contented with recommending his views to the close attention of our readers and referring them for comparative illustration to the work of Reissessen, and the paper recently published by Mr. Addison in the Transactions of the Royal Society on the minute structure of the lungs, to the article Hemoptysis in the Cyclop. of Pract. Med. by Dr. Law, and Dr. Townshend's Essay on Pulmonary Apoplexy. We have already stated that in cases of pulmonary apoplexy no hesitation should be experienced in the employment of free depletion; after this has been practised, the remedy in which Dr. Graves reposes most confidence is ipecacuanha given in doses of "two grains every quarter of an hour, until there is some improvement, and then every half hour or hour, until the bleeding stops." We are indebted to Richter for first making known the powers of ipecacuanha in restraining hemorrhage, the effects of the remedy being not confined to hemoptysis. Dr. Graves recommends that its employment be preceded by a purgative enema, and a saline cathartic, and states that it may be given with advantage in hemorrhage from the bowels, and even in hematemesis, he preferring it to the acetate of lead, which he employs only in those cases of passive hemorrhage in which opium is indicated, and then in combination with the last remedy.

Phthisis. In the following passage the author explains his views on the pathology of tubercle:

"I look on tubercular development and consumption as the consequences of that particular state of constitution, which occasions what is falsely termed tubercular inflammation, a state of constitution in which we have three distinct processes, attended by corresponding morbid changes, each different in itself, but depending on one common cause. Every form of consumption, which has hitherto come under our notice, is referrible to one common origin, and this is that debilitated state of constitution which has been termed the scrofulous habit. One of the first tendencies of this habit is to the formation of tissues of an inferior degree of animalization, among which I class tubercles, whether occurring in the lungs, brain, or liver, whether they exist in a minute or granular form, or in large, soft, and yellow masses, or in the state of tubercular infiltration. I look on them as one of the first of these morbid changes dependent on a peculiar constitution of body, and most commonly found to accompany it. The weaker the constitution is, the greater tendency is there to generate tissues of a lower degree of vitality, and, on this principle, I think we can explain the occurrence of entozoa and hydatids. There are some cases in which you will never be able to prevent the generation of intestinal worms, until you direct your attention to the source of the evil, which lies in the weakness of the constitution, for, in such a state of the system all animals are liable to the formation of parasitic productions and tissues imperfectly animalized. I look on tubercles in this light, and not as the consequence of inflammation, nor do I consider that it has been proved that tubercular development is the cause of phthisis." (pp. 279.)

However this may be, and we are not among those who feel disposed to dispute much of what is now advanced, there can be no question that the presence of tubercles in the lungs, forms a most important element in a large majority of the cases of phthisis which we are called upon to treat, and the effect of masses of tubercular infiltration, or of numerous isolated tubercles scattered throughout the pulmonary tissue upon the functions of the lungs, independent of the question of subsequent irritation arising from their presence, cannot be otherwise than of the most injurious character. Dr. Graves has himself seen tubercles to an extraordinary extent make their appearance in the lung in the space of two or three weeks, and has known persons to die of the suffocation caused by this rapid development without the usual symptoms of phthisis; and it must have occurred to many to observe cases in which a similar rapid and fatal change takes place from recent deposition where the symptoms had indicated that a small extent only of the pulmonary tissue had been previously implicated. It may indeed be stated as a general principle that the first deposit of tubercle is rarely to such an extent, as to produce fatal effects, although it can scarcely be questioned that where tubercular deposition has at any time taken place, so as to become appreciable, the predisposition to further deposit is most fearfully increased. The most important, then, and, as Dr. Graves observes, one of the first morbid changes arising from the scrofulous habit, is the formation of tubercular matter. The presence of tubercles in any of its forms, however, is not so much the disease we are called upon to combat as one of its effects.

Dr. Graves contends that in all cases of phthisis "the pectoral symptoms, of whatever nature they may be, are caused by scrofulous inflammation," by which we presume that he means, inflammation as it occurs in individuals of a scrofulous diathesis, and he proceeds to compare the progress of ulcerations of the lungs with that of external scrofulous abscesses. There is, he observes, the same slowness, the same insidious latency, the same gradual solidification and gradual softening; the puriform fluid secreted is similar in characters, while there is the analogous occurrence of burrowing ulcers and fistulous openings with close approximation in the form of thin parietes, and difficulty of healing in each; and at the same time constitutional symptoms identical in nature; hectic flushings and sweats, diarrhœa, emaciation, &c. equally accompany phthisical suppuration of the lungs and scrofulous inflammation of the joints or other external parts. With these views, therefore, we are not surprised to find Dr. Graves entertaining the opinion that tubercle, though a most frequent accompaniment of phthisis, is neither the essential cause of that disease nor a necessary product. Scrofulous inflammation is with him the *fons et origo*, the real and efficient cause of phthisis, whether tubercle be generated in the course of the diseased action or no, and thus we have scrofulous pneunomia and scrofulous bronchitis equally productive of phthisis without the presence of one single tubercle or spot of deposition of tubercular matter, either in the pulmonary tissue or on the bronchial membrane. In the latter case, scrofulous bronchitis, it is urged by Dr. Graves, that the accompanying fever presents all the material phenomena of phthisis; there is the same emaciation, frequently the same incurability; the same means tend to its aggravation or benefit, and the same scrofulous pus is secreted although not mixed as in cases of true phthisis with broken-down tubercles.

But if we are to admit of this line of argument we might include chronic pleurisy and chronic pneumonia occurring in scrofulous constitutions, with many other forms of pulmonary disease in the same category. The question after all is merely one of name, Dr. Graves understanding the term phthisis in rather a wider and, perhaps it may be said, more indefinite sense, than as it is now generally received. Still, however, if terms are signs by which corresponding ideas are to be conveyed, the more definite their use the more accurate the knowledge derived from them; and though we are by no means disposed to question the fact that tubercle is not an inseparable accident of scrofulous inflammation, the constitutional symptoms of which are the same whatever organ is affected, and the more prominent local symptoms of which, where the pulmonary tissue or bronchial membrane is affected, are the same with those in which there is tubercular deposition,—still we think the term phthisis, as used by modern authorities to indicate tubercular disease of the lungs, has its convenience, and we should be sorry not to retain it in that signification.

That inflammation of the bronchial membrane occurring in persons of marked scrofulous constitution may assume a peculiar character, we see no reason to doubt, and we are quite as willing to admit that there is a form of bronchial disease to which the term scrofulous bronchitis may be applied with as much propriety as the term scrofulous ophthalmia is applied to the inflammations of the eyelids and conjunctiva, of such frequent occurrence in like constitutions. It may also be admitted that the bronchial affections, so commonly met with in tubercular phthisis, are of this character, and that such affections, while, as is well known to most practitioners, they give rise to an increase of cough, dyspnea, and other among the most distressing symptoms of the disease, may also have much to do in producing the chills, flushings, perspirations, and other signs of constitutional disturbance. On the other hand, it by no means follows that tubercle is otherwise connected with them in its origin than as taking its rise from the same constitutional taint or deviation from the healthy standard, predisposing to a certain series of morbid actions in those exposed to causes of disease, rather than to a certain other series which would be developed in constitutions of a different character.

In persons of the scrofulous diathesis every disease assumes more or less of a scrofulous nature, one of the main characteristics of which is a tendency to the deposition of tubercles. But, as it seems to us, these are aberrations of the nutritive and assimilative functions which may or may not occur, giving rise by their presence in whatever organ to serious disease, predisposing to attacks of inflammation, which in such cases assumes the scrofulous character, and sometimes taking place in immediate connexion with but not necessarily dependent on such inflammation. When such a deposition takes place in the lungs, we have the disease to which, by almost universal consent, the term phthisis is now assigned, among the primary symptoms of which are found more or less of cough, dyspnea, quickened circulations and other signs of local and constitutional irritation varying according to the extent of the deposition. To these a disposition to bronchial congestion is added, necessarily connected as it would appear with the diminished capacity of the pulmonary tissue. Frequent attacks or paroxysms of bronchial disease, scrofulous bronchitis if you like, are the consequence, and now we have the more marked characteristics of phthisis in its advanced stages, hectic, chills, evening flushings, and night sweats, purulent expectoration, &c., but in all this we see tubercles as the primary disease, certainly as a prior link in the chain to scrofulous bronchitis, and which therefore we look upon as the true pathological character of phthisis, although perhaps most of the leading symptoms in its later stages may be identical with those observed in other affections, occurring under similar constitutional peculiarities. " But," says Dr. Graves, " if tubercles were capable of producing inflammation, we should discover some traces of it in every lung where they are found to exist, and yet you will meet many cases in which you cannot detect the slightest trace of it down to the very edge of the tubercular mass." (p. 282.) We can by no means agree in this conclusion; various circumstances may occur to restrain or counteract the influence of any active cause of disease, to become a preventive check upon its energy, or to neutralize its effects. That tubercle is per se necessarily productive of inflammation is another affair, but we cannot doubt that it may and does frequently prove the cause of the inflammation and congestion of the bronchial membrane in the lungs of those affected with it. Upon the whole we are disposed to admit the three forms of disease in the lungs recognized by Dr. Graves, as arising from scrofula—scrofulous pneumonia, scrofulous bronchitis, and tubercular development.

" We may, therefore, have tubercles without either the pneumonia or the bronchitis; and we may have scrofulous pneumonia often ending in slow burrowing suppuration, and proving fatal without any tubercles being formed. In like manner, a person may die of scrofulous bronchitis without the occurrence of either tubercles or pneumonia. Of these three effects of scrofula, it may be remarked, that, owing to their cause and origin being the same, they are most frequently found in combination. The same diathesis which produces one may give rise to the others; hence the frequency of their association; hence it is that they generally occur together." (p. 282.)

The last of the three, tubercular development, is distinctly recognizable, in the greater number of instances, from the other two by careful auscultation, and although many of the symptoms, constitutional and local, may be common to all, it is right that it should be as distinctly marked as a separate disease.

The remarks of Dr. Graves on the treatment of phthisis and of persons in whom phthisis threatens are judicious and confirmatory of the views of the best practical authorities upon the subject. Viewing the disease in all its forms, as taking its origin in a scrofulous tendency, his prophylactic measures are directed to the strengthening and support of the general system and the improvement of its tone. Early rising, cold washing, free exposure to the air, exercise, with nutritious not stimulating diet, form the main point of the regimen which he recommends. The treatment of persons in whom tubercle has become developed in the lungs, or who are attacked with scrofulous bronchitis or pneumonia, is conducted upon the same principles as those which are recognized in inflammation of other organs occurring in scrofulous constitutions. It is with regret that we are compelled to add that little if anything is contributed to what is already well known, and no additional prospect given of effecting the cure of consumption.

The proposal made in a subsequent lecture to cure certain forms of phthisis by rapid mercurialization, may be thought by some to prove an exception to this remark, and though our readers are probably already acquainted with the views of Dr. Graves and others upon this point, we cannot, therefore, altogether pass it over without notice. The idea seems to have occurred about the same time to Sir Henry Marsh, Dr. Graves, and Dr. Stokes, and originated, at least as far as Dr. Graves is concerned, in the success attending Dr. O'Beirne's employment of the same means in acute scrofulous inflammation of the joints. The form of phthisis in which this practice is stated to have proved successful in effecting a cure, is where the lungs become affected before any general contamination of the system takes place. "It is in such cases, and such only," says Dr. Graves, "that mercury ought to be tried, and it will avail nothing except when the commencement of the scrofulous inflammation of the lung has arisen suddenly, and in consequence of the operation of some obvious cause, as catching cold or the occurrence of hemoptysis." (p. 602.) Some instances are related of the good effects of this plan of treatment, and the observations of Dr. Munk on the same subject quoted at length from the Medical Gazette. The practice is not altogether new, but has been before recommended and followed by Dr. Rush and some other American physicians. They, however, failed to indicate the class of cases in which it was calculated to prove of service, and the indiscriminate employment of the method, and its consequent want of success in a vast number of instances may probably have been the cause of its having fallen into disuse. In the cases in which the mercurial treatment proves applicable, the tubercular affection, however, would seem to be of only secondary importance, so that this method appears to be chiefly serviceable in scrofulous pulmonary inflammation, rather than in tubercular phthisis, affording an additional reason derived from curative indications for retaining the term phthisis in its more definite sense.

Enlargement of the liver. In lecture thirty-six we meet with some interesting observations on the occurrence of hypertrophy of the liver from various causes, and the occasional curability of this affection. In persons below thirty, it is observed, the liver may become very considerably enlarged and yet under proper treatment return again to its natural size. Several cases are referred to in illustration and among them one remarkable instance of an old gentleman between seventy and eighty years of age who had enormously enlarged liver and ascites, in which the enlargement rapidly decreased with a corresponding improvement of the general health under the use of blue pill in combination with hydriodate of potash.

The enlargement of the liver here spoken of, Dr. Graves traces to a cachectic habit, and regards it as similar to what is frequently observed in scrofulous habits and in persons suffering under the conjoined influence of the poison of syphilis and the injudicious use of mercury. When it was the custom to salivate every venereal patient and to keep him under full mercurial influence for a month or two, it frequently happened, says Dr. Graves, that just as the mercurial course was finished the patient got disease and enlargement of the liver. It is hinted that some connexion might perhaps be traced between the stimulant effects of mercury on the liver and the subsequent hypertrophy. Dr. Graves, however, for the present contents himself with noticing the fact; leaving, as he says, the explanation to his juniors, who always explain matters, according to his observation, much more readily than their seniors. Other diseased states to which hypertrophy of the liver is traced as one of the results of a general affection of the system are scarlet fever, morbid conditions of the heart, and intermittent fevers. It is not however necessary that we should in this place examine the subject at greater length.

Paraplegia. It is well known that much obscurity hangs over the precise seat of many cases of paraplegia, and the cause of the symptoms has been variously located by different observers and in different instances of the disease, according to the general views taken by the observer or the peculiar features of the individual case. The instances of paraplegia to which Dr. Graves directs attention in his thirtieth lecture differ from those commonly described as arising from morbid action in some part of the cerebro-spinal axis in several important particulars. Two forms are especially particularized, and the cases of either form resemble each other in taking their origin from impressions primarily made upon the peripheral extremities of the nerves. In the form first described these impressions would seem to be conveyed by reflex action to the medullary column from various parts of the system, subsequently giving rise to paralysis of the lower extremities. Instances are related in which the mucous membrane of the intestinal canal, the liver, and other abdominal organs were the parts primarily affected; in others, mentioned by Mr. Stanley and Dr. Stokes, disease of the kidneys was the apparent cause of the subsequent paraplegia. In several of these cases the spine was carefully inspected and no morbid change, after the most patient scrutiny, could be detected. Dr. Graves correctly observes that examples are not wanting of paralysis of other parts of the body produced in like manner, and refers also to the effects on the nervous system of another character produced by the reflex action of irritating impressions on the intestinal mucous membrane, as tending to explain these cases. Thus convulsive paroxysms are very commonly caused by the presence of worms in the intestines and by the accumulation of irritating matters of various kinds.

The following example of a partially paralyzed state of the retina, apparently depending upon an impression made upon the cutaneous branches of the nerves of the face, is interesting:

"A medical student, travelling through Wales on the outside of the mail, was exposed for many hours to a keen north-easterly wind blowing directly in his face. When he arrived at the end of his journey he found that his vision was impaired, and that everything seemed as if he was looking through a gauze veil. There was no headach, no symptom of indigestion, to account for this evidently slight degree of amaurosis, and yet he was recommended to use cupping at the nape of the neck and strong purgatives. When he consulted me, which he did in the course of a few days afterwards, I at once saw that there was something unusual in the case; and, after a careful examination, I at length elicited from him the fact of his having been exposed to the influence of the cold wind. It was now apparent that the retina suffered in consequence of an impression made on the facial branches of the fifth pair. The cure was effected, not by a treatment directed to relieve cerebral congestion, but by stimulation of the skin of the face, forehead, temples, &c." (p. 398.)

In some of the cases referred to, especially those which are connected with and were the sequelæ of fever, the spinal cord may have been itself primarily affected, and the absence of appreciable lesion on inspection forms no conclusive argument to the contrary. Still we are not disposed to question that his views with respect to the occurrence of this and other forms of paralysis as well as other affections of the nervous system from a morbid state of the peripheral extremities of the nerves, are in the main correct. With the remarks which follow we perfectly coincide:

"I could give many instances," says Dr. Graves, "of pains commencing in particular parts of the body and travelling back towards the spine so as to give rise to an affection of that organ, which has been too generally looked upon as the result of idiopathic disease. How often does this happen in hysteria? How often does it occur that the organ primarily engaged in hysterical cases becomes during the attacks acutely painful, and as the disease proceeds the pain travels back towards the spine, until at length the spinal cord itself becomes affected and we find acute pain and tenderness over some portion of its track? I am fully persuaded that many modern authors who have ascribed the phenomena of hysteric and other affections to spinal irritation, have been too hasty and indiscriminate in their explanations. In the majority of cases you will find hysteria patients complain at first, not of pain in any part of the spinal cord, but in the right side in the situation of the liver, in the region of the heart or stomach, or in the head, or the pelvic region. At this period there is seldom any tenderness over the spinal cord; but as the disease goes on the irritation which existed in some of those situations to which I have referred is extended to the spine, and pain and tenderness are now felt over some of the spinous processes of the vertebræ. When this has taken place, then the spinal irritation thus produced becomes itself a new cause of disease, from which, as a centre, the morbid influence is propagated to other organs." (p. 400.)

The other form of paraplegia described by Dr. Graves, and which he is certainly mistaken in believing not to have been mentioned by any other writers, also arises from affection of the extremities of the nerves. In this form, however, there is no reflex action, but the paralysis is apparently the result of a direct impression made upon the nerves of the affected limbs. The following case is an example:

"James Moore, aged 32, was admitted into the Meath Hospital on the 3d of March, 1833, under Dr. Stokes's care, for an attack of paraplegia, which he attributed to cold and wet feet while engaged in a quarry. About a month before admission he perceived a stiffness of the great toe of his right foot, afterwards numbness and coldness of the sole, and then of the leg as far as the knee, and dragging of the limb in walking. During the progression of the disease up along the right thigh it commenced in the left foot, and after a few days he experienced almost complete paralysis of sensation in the right lower extremity, and a lesser degree in the left, accompained by so much diminution of the power of motion, as to render him unable to walk without support. About three weeks after the appearance of paralysis in the lower extremities, the little finger of the right hand was attacked with numbness, which passed successively to the rest, attended by some loss of the sense of touch and power of grasping objects. He had also retention of urine, and the bowels were obstinately constipated. There was no tenderness over any part of the spine. He had no pain in the head; his pupils were natural; pulse, sleep, appetite also normal." (p. 415.)

Dr. Graves relates several cases of this description; others must have occurred to the notice of such as are engaged in extensive practice. The main feature of this form of paralysis would seem to be loss of power or sensation or both in the extremities of the nerves distributed to the surface, arising from a direct morbid impression made upon them, and sometimes gradually creeping along their course in a retrograde direction, thus implicating other parts in succession, and by extension of the morbid impression, to the spine, gradually becoming propagated to more remote parts of the system. This description of paralysis seems very generally to arise from exposure to cold and wet, two instances of which, when the affection has continued purely local, we have ourselves witnessed. In both of these cases the hands had been kept partially immersed in cold water during many hours, and in both there was loss of power and sensation, which, although it did not spread, resisted every measure which was employed for relief. In one case the paralysed state of the hands still continues, the other we have lost sight of.

Similar to these, though arising from a different cause, would seem to be the dropped hands of the painters. The loss of power in these cases appears as often to arise from the direct impression made by the lead poison on the extremities of the nervous fibriles distributed to the hands, as from the absorption of the poison within the system. The paralysis arising during the course of painters' colic might be attributed to a reflex action on the muscular system, owing to the impression made on the intestinal canal, as in the form of paraplegia first alluded to, but Dr. Graves is disposed to think that it results rather from the direct impression of the poison on the nervous system, and he is probably right.

Before leaving this subject we may allude to another diseased condition in which the spinal cord becomes affected, and paralysis is ultimately generated from impressions originally made as it would seem upon the peripheral extremities of the nerves. This is pointed out by Dr. Graves in a subsequent lecture, where he endeavours to establish the point, "that in certain cases where gout attacks the nerves, giving rise to gouty congestion or inflammation, frequently recurring, and acquiring increased strength, and deeper root as it proceeds, the morbid affection may, after years or even months, run on until it reaches the spinal cord, involving a certain portion or portions of that organ, and producing loss of sensation and motion commensurate to the amount of spinal derangement," (p. 588;) or, as he otherwise lays it down, "*that gouty inflammation of the nerves and their neurilema may, in process of time, extend to the*

*spinal marrow and its investments, and give rise to derangements of the latter, terminating in ramollissement and structural derangement*, (p. 589.) The principle here attempted to be established is illustrated by some important cases, and gives rise to the practical indication of endeavouring to anticipate and divert the attack elsewhere, when the cerebro-spinal axis is threatened. For this purpose Dr. Graves, having experienced the total inefficacy of colchicum, hydriodate of potash, and all the usual remedies for gout in relieving or removing this form of the disease, recommends the early insertion of issues over the spine with prompt and decided mercurialization.

Inflammation. The thirty-third lecture is devoted to the consideration of inflammation, and the motor power of the circulation. Both of these subjects, at least in the mode in which they are treated, are foreign to the professed objects of the work, while it is sufficiently evident that to take such a view of either of them as would suffice to come to a clear understanding, would far exceed the limits which we could at present spare for the purpose. With respect to the latter subject therefore, "the motor powers which cause and regulate the circulation," we must content ourselves with stating that Dr. Graves is of opinion that the capillary vessels play a most important part, and quotes with approbation the views put forth by Dr. Carpenter in his Physiology.

In inflammation all is attributed to the active force of the capillaries. "*The capillaries have the initiative ; with them commences the enlargement which afterwards extends to the smaller arteries, and from these to the larger branches.*" (p. 484.) Dr. Graves is opposed to the idea that in this enlargement there is, as was contended for by Dr. Hastings and Dr. Wilson Philip, any traces of debility. He says distinctly that the swelling, exaltation of nervous sensibility, increase of temperature, and augmented secretion, are all in fact opposed to the theory of debility. "There is no passive dilatation from weakness, the capillaries enlarge from increased and not from diminished action : red blood finds its way into vessels which before received only white, and unusual secretions occur in the affected parts." Now though we have not space to consider and perhaps are not inclined altogether to deny what Dr. Graves advances, as to the property which the capillaries possess of actively dilating and drawing the blood into them being one of the principal causes of the circulation, we can by no means admit that the property in question has been so far shown to appertain to these minute vessels as to entitle it to be received as the basis of pathological deduction. Dr. Hastings observed certain facts in the capillary circulation which, as he conceived, admitted of explanation upon the principle of debility. Whether the stagnation of the blood in the irritated and inflamed capillaries, and the distended state of these vessels observed by him, were caused by debility of their coats or not, there is no question that the distention, or dilatation if you will, does take place, and that, from the crowding of the blood-corpuscles, there is subsequent obstruction. If the active power of the capillaries be the sole efficient and continuing agent of this influx of red globules, there seems to be no reason why these minute vessels should retain the globules imprisoned within their embrace for an indefinite period of time, and at great injury to the vital organization of the part.

Without entering into the controversial discussion which the author takes up with Müller, Dr. Marshall Hall, Dr. C. Williams, and others, and without pledging ourselves to the opinions advanced by these authorities, we cannot but remark that as far as relates to the expression of the fact, Dr. Hall is correct when he states that the first appreciable physical effect of the inflammatory process is the adhesion of the blood-corpuscles upon the internal surface of the capillaries, which is followed by their ultimate stagnation. Upon the statement of this fact, as made by Dr. Hall, we find the following remark:

"Here you perceive that the first step is the adherence of the globules of the blood to the internal surface of the capillaries, the consequence of which is that the caliber of these vessels is considerably diminished so that they become obstructed and cause a stagnation of the blood, which Dr. Hall looks upon as the essential character of inflammation." (p. 466.)

But this is a very unfair inference, and one that is by no means borne out by facts which Dr. Graves may, if he please, observe. It has been shown by Mr. Addison, and we have ourselves verified the observation, that in the passage of the blood through the capillaries the corpuscles pass in single file, some of these occasionally turning aside, as it were, from the current and for a time attaching themselves to the parietes of the vessel, those succeeding passing round or rolling over them as they partially project from the walls. Now in the inflamed or irritated state of the same capillaries, the corpuscles rush in greater numbers through the vessel, the caliber of which is not "considerably diminished," but on the contrary considerably increased, the globules passing in double and triple files, or crowding in still greater numbers with the current of fluid, while the sides of the vessel are occupied by numbers of them in a state of stagnation, some of which from time to time roll off into the general current, others from behind coming up and taking their places, until at length, whether from over distention or debility, scarcely we think from active dilatation, and certainly not from contraction or diminution of caliber, the vessels become obstructed and there is eventually a complete stagnation.

But Dr. Graves and others have reasoned upon the view that the capillaries are simple membranous tubes. We question whether it has yet been proved that such is their nature, or, indeed, whether they are anything more than interstitial passages. We cannot, however, enter into the subject here. Our readers will find many facts bearing upon the investigation in Mr. Addison's paper just published in the eleventh volume of the Provincial Medical and Surgical Transactions.

There are several other subjects we had marked for consideration, and to which we should have much wished to direct attention, but to give even an imperfect abstract of all that is worthy of notice in a work such as that which Dr. Graves has produced is obviously impossible. Among those which we are compelled to pass over are the observations on syphilitic diseases, and many important points connected with them. These occupy lectures twenty-three to twenty-nine inclusive, and would alone require a special review to do justice to them. In lecture thirty-one we find a notice of the successful employment, at the Meath Hospital, of electro-magnetism, in various affections, by Mr. Clarke. The diseases in which this agent proved serviceable are chronic rheumatism, lumbago, and sciatica, neuralgia, amenorrhea, and irregularities of the catamenia and their sequelæ, amaurosis, deafness, some forms of paralysis, especially those termed partial paralysis, and various nervous, neuralgic, and rheumatic pains. These, it will be observed, are precisely the class of cases which are relieved by electricity employed in other modes, but there would seem to be advantages attending this peculiar method of generating it. In the succeeding lecture on sleeplessness, there are some excellent observations, and it is stated that in the form which frequently occurs in hysterical female hypochondriacs, and persons of nervous and irritable habit, Dr. Graves has found antispasmodics, musk, and assafetida serviceable, when opium in all its forms, and all kinds of narcotics, had previously failed. The cases of jaundice appended are interesting, and show the great importance of immediate attention being paid to nervous symptoms, arising in the course of that disease. Other subjects on which there are valuable remarks scattered through various lectures, are erysipelas, phlebitis, glanders, phlegmasia dolens, cancrum oris, various diseases of the skin, diarrhea, amaurosis, periostitis, &c. Our favorable opinion of the entire work may be gathered from the preceding remarks. We cannot exactly call it, with the author, a System of Clinical Medicine, but no practitioner of medicine should be without it, since there is scarcely a disease to which the human frame is liable which does not receive in it some illustration, direct or incidental; and, as a guide to practice, in very many instances, especially when difficulties arise, it will often be found a most useful work for reference.

---

